# Methamphetamine-induced encephalopathy in the absence of hyperammonemia

**DOI:** 10.1186/s12888-023-04764-2

**Published:** 2023-04-20

**Authors:** Jessica M Rabbany, Kaylee Fitzgerald, Jade Bowman, Fanglong Dong, Michael M. Neeki

**Affiliations:** 1 Department of Behavioral Health, Arrowhead Regional Medical Center, Colton, CA USA; 2 Department of Emergency Medicine, Arrowhead Regional Medical Center, Colton, CA USA; 3 California University of Science and Medicine, Colton, CA USA

**Keywords:** Methamphetamine, Encephalopathy, Hyperammonemia, Altered mental status, Neurotoxicity

## Abstract

**Background:**

Methamphetamine is an addictive drug with various effects on the neurotransmitters in the central nervous system. Methamphetamine-induced encephalopathy in the absence of hyperammonemia presents a unique challenge in a clinical setting. Previously published cases of methamphetamine-induced encephalopathy suggested that methamphetamine-induced hepatotoxicity and subsequent hyperammonemia may be the cause of encephalopathy. However, the literature is limited on methamphetamine-induced encephalopathy without hyperammonemia.

**Case:**

This case presents a disoriented patient with methamphetamine use disorder in acute toxicity, unable to ambulate independently, and poorly responsive to verbal stimuli. The patient was found to have normal ammonia levels.

**Discussion:**

This patient’s presentation and laboratory findings, namely normal ammonia levels, suggest a different pathophysiological pathway for methamphetamine-induced encephalopathy. One potential pathway is through the direct action of methamphetamine on the central nervous system through acute disruption of neurotransmitter signaling and disruption of the blood-brain barrier.

**Conclusion:**

Further research should be conducted into the prevalence and pathophysiology of methamphetamine-induced encephalopathy in the absence of hyperammonemia.

**Key Points:**

Methamphetamine-induced encephalopathy (MIE) in the absence of hyperammonemia presents a unique challenge in a clinical setting.

Previously published cases of MIE suggest that methamphetamine-induced hepatotoxicity and subsequent hyperammonemia may be the cause of encephalopathy.

Further research should be conducted into the prevalence and pathophysiology of MIE in the absence of hyperammonemia.

## Background

Methamphetamine is an addictive stimulant that affects the central nervous system (CNS) [1]. It increases the levels of dopamine in the brain which reinforces the dependency on the drug [1]. The Centers for Disease Control and Prevention reported that about 1.6 million adults in the United States used methamphetamine annually between the years of 2015–2018, and 25% of those reported using injectable forms [2]. Higher rates were seen among men aged 26–34, followed by 18–25 age groups [2]. Methamphetamine use has increased by 43% and frequent use of the drug (defined as using for at least 100 days in the past year) increased by 66% between the years of 2015–2019 [3]. In 2020 alone, the National Survey of Drug Use and Health reported that 2.6 million people (0.9% of total population) in the United States aged 12 and older used methamphetamine in the past year [3]. Despite these increasing numbers, less than one-third of the users received substance use treatment in the past year [2].

Toxic doses of methamphetamine can lead to the development of encephalopathy by various mechanisms. The National Institute of Health defines encephalopathy as any diffuse disease of the brain that alters brain function or structure [4]. Symptoms may range from subtle changes in personality to progressive loss of consciousness. However, the hallmark symptom of encephalopathy is an altered mental state [4]. There are many mechanisms that may lead to a patient developing this problem, including but not limited to, metabolic, anatomic, traumatic, shock, infectious, and toxic. Metabolic causes are varied and can be secondary to lack of oxygen, glucose, metabolic cofactors (i.e., vitamin deficiencies), or peripheral organ dysfunction, such as hepatic, uremic, or dialysis-induced encephalopathy [5]. Even inherited diseases and some neuroendocrine disorders can eventually disrupt normal brain function and lead to encephalopathy [6].

One of the suggested causes of encephalopathy is of hepatic origin. There are approximately 7–11 million cases of hepatic encephalopathy in the United States, with 150,000 new diagnoses each year [5]. Most cases are secondary to advanced liver disease, either acute or chronic. Triggers in the setting of chronic liver disease are extensive and include infection with hepatitis C, gastrointestinal bleeding, constipation, renal failure, electrolyte imbalances, diuretic overdose, and substance use such as alcohol [5, 7]. Under normal conditions, the liver works to metabolize and clear ammonia from the blood, however, in a diseased state, it is unable to perform this function. This accumulation of ammonia in the blood, termed hyperammonemia, can cause neurotoxic effects once it crosses the blood-brain-barrier (BBB), leading to encephalopathy [5, 8]. Here we present a male patient with possible methamphetamine-induced encephalopathy (MIE) due to methamphetamine toxicity. The exact incidence of MIE is not known and this case will add to the current body of literature.

## Case

A 47-year-old male brought into the hospital by a local law enforcement agency for evaluation of inability to care for himself. Patient was initially apprehended after a brief scuffle and exposure to pepper spray by the deputies for resisting arrest. He was charged with possession and use of methamphetamine and was transferred to a local detention center. Upon evaluation by the detention medical staff, the patient was only alert and oriented to self, unable to engage in meaningful conversation, and unable to ambulate without assistance. As a result, the patient was placed on an involuntary 72-hour hold for grave disability and transferred to the local hospital for medical and psychiatric evaluation.

On arrival at the behavioral health unit of the hospital, he was not oriented to person, place, or time. He was repeatedly mumbling numbers, possibly a phone number, without clear context. He was unable to ambulate independently and was poorly responsive to conversation, with moderate response to painful stimuli. His initial vitals included blood pressure of 181/129 millimeter of mercury (mmHg), heart rate of 98 beats per minute, temporal temperature of 36.5 °C (97.7 °F), respiratory rate of 34 breaths per minute and oxygen saturation (SpO2) of 100% in the room air. Bedside serum glucose was also checked and found to be within normal limits. Because of the complexity of his presentation on arrival, he was immediately wheeled to the main emergency department (ED) for further medical evaluation.

In the main ED, his physical examination was remarkable for ill-appearance, disorientation, tachycardia, respiratory distress, and musculoskeletal weakness. Patient had old and new abrasions bilaterally on his upper and lower extremities. A deformity was noted on his right wrist. His past medical history in the electronic health records was significant for rhabdomyolysis, history of methamphetamine use disorder. Shortly after arrival at the ED, his mental status deteriorated, the patient was noted to have frothy white secretions from his mouth and poor gag reflex. He was subsequently intubated for airway protection and placed on a ventilator.

**Fig. 1:**
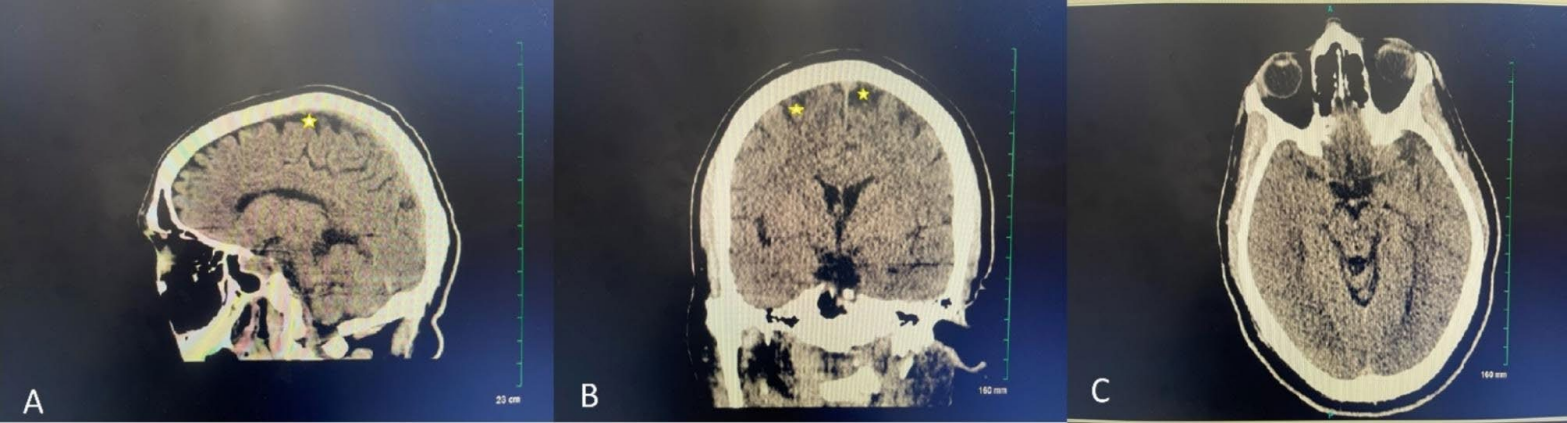
1 A Sagittal, 1B Coronal, and 1 C Axial Non-contrast CT of the brain indicating mild age-related atrophy (stars) without any acute abnormalities

The laboratory analyses were significant for leukocytosis of 18,800 cells per microliter, blood urea nitrogen of 36 milligrams per deciliter (mg/dL), creatine kinase of 6,998 units per liter, creatinine of 2.28 mg/dL and urine drug screen was positive for methamphetamine (Table 1). The liver function tests were slightly abnormal; however this is consistent with this patient’s known history of concurrent alcohol use. Ammonia level was found to be 71 micrograms per deciliter (ug/Dl) which is within normal limits (20–80 ug/Dl). In addition, the patient underwent a computerized tomography (CT) scan of his brain without contrast that showed mild age-related atrophy but failed to show any acute abnormalities. (Fig. 1).

Subsequently, patient was admitted to the hospital with diagnosis of rhabdomyolysis, respiratory failure, and acute kidney injury. He was treated with intravenous lactated Ringer at 150 milliliters per hour continuously. Concurrently, he was psychologically stabilized with Abilify 5 mg daily. After two days on the ventilator support and sedation, He was extubated, and his physiological/mental status improved. Further consultation regarding his substance use disorder along with his social status was initiated and the patient was discharged to supportive outpatient care without further incident.

**Table 1:** Laboratory findings including complete blood cell count (CBC) with manual differential, venous blood gas (VBG), and complete metabolic panel (CMP).

Complete Blood Count	Value	Reference Range
White Blood Cell Count	18.8	4.5–11.1 10^3^/microliter
Red Blood Cell Count	5.04	4.50–5.90 10^6^/microliter
Hemoglobin	16.1	13.0–17.0 gram per deciliter
Hematocrit	47	41–53%
Mean Corpuscular Volume	94	80.0–100.0 femtoliter
Mean Corpuscular Hemoglobin	31.9	26.0–32.0 picogram
Mean Corpuscular Hemoglobin Concentration	34	33–35 gram per deciliter
Red Cell Distribution Width	14	11.0–15.0%
Platelets	252	120–360 10^3^/ microliter
**Manual Differential**		
Neutrophils %	88	
Lymphocytes %	4	
Monocytes %	5	
Eosinophils %	0	
Basophils %	0	
Bands %	3	
Absolute Neutrophil Count	17.1	10^3^/microliter
**Venous Blood Gas**		
pH, Venous	7.36	7.35–7.45
pCO2, Venous	33	35–45 mm of mercury
HCO3, Venous	18.4	20–24 millimoles per liter
Base Excess, Venous	-5.9	-2.0–2.0 millimoles per liter
O2 Content, Venous	20.3	
Blood Gas Hemoglobin	16.9	13–17 gram per deciliter
pO2, Venous	53	30–50 mm of mercury
Potassium, Blood Gas	3.95	3.5–5.5 millimoles per liter
Chloride Blood Gas	104	98–106 millimoles per liter
Lactate, Venous	2.74	0.5–2 millimoles per liter
Oxygen Saturation	100	
Sodium, Blood Gas	142.1	135–148 millimoles per liter
**Complete Metabolic Panel**		
Sodium	137	135–148 millimoles per liter
Potassium	4	3.5–5.5 millimoles per liter
Chloride	101	98–110 millimoles per liter
CO2	17	24–34 millimoles per liter
Blood Urea Nitrogen	36	8–20 milligram per deciliter
Creatinine	2.28	0.50–1.50 milligram per deciliter
Glucose	165	65–125 milligram per deciliter
Calcium	9.5	8.5–10.5 milligram per deciliter
Total Bilirubin	1.2	0.0–1.2 milligram per deciliter
Alkaline Phosphatase	115	35–125 µl
Aspartate Transferase	137	5–40 µl
Alanine Transaminase	43	5–40 µl
Total Protein	8.4	6.0–8.0 gram per deciliter
Albumin	4.6	3.5–4.9 gram per deciliter

### Follow-up

Since this admission one year ago, this patient has been seen in the hospital and behavioral health unit four more times. Currently, there is poor control of his psychiatric conditions due to continuous use of methamphetamine, lack of follow-up for medical and psychiatric care, and lack of social support. Each admission showed laboratory and diagnostic imaging results consistent with his initial presentation including normal ammonia levels and slightly elevated liver function tests.

## Discussion

The differential diagnosis for cases of encephalopathy may include shock, anatomical, metabolic, toxic, and infectious etiologies. His leukocytosis with neutrophil predominance could suggest acute infection. However, this was ruled out by thorough infectious workup, including blood and urine cultures, lactate levels, urinalysis, chest x-ray, and complete skin inspection. Previous studies noted that patients with methamphetamine toxicity had significantly higher white blood count levels, with no evidence of infectious or inflammatory disease, when compared to their control cohort [9, 10]. The patient in this case had a well-documented history of methamphetamine use disorder, and his urine drug screen was positive for methamphetamine. As a result, his transient encephalopathy, presenting without any evidence of anatomical trauma, infection, metabolic abnormalities, or shock, could more likely be a result of methamphetamine-induced CNS toxicity. Due to initial findings of elevated blood urea nitrogen, creatinine, and creatine kinase levels, other possible causes of encephalopathy were investigated during his hospital course of stay.

Classically, MIE has been explained by elevated serum ammonia levels [11]. Treatment with lactulose subsequently improved mental state in those patients, suggesting that their encephalopathy was likely related to hyperammonemia. In a rodent study, Halpin and colleagues noted that plasma and brain ammonia levels were significantly elevated two hours after administration of methamphetamine [12]. Furthermore, the administration of lactulose blocked this increase in ammonia levels [12]. As a result, the authors hypothesized that the mechanism for elevated ammonia after methamphetamine exposure is methamphetamine-induced hepatotoxicity, impairing ammonia clearance from the blood [12]. However, the patient in this case had normal serum ammonia levels, suggesting there may be an alternative pathophysiological mechanism, including the effect of methamphetamine on neurotransmitter levels in the CNS, methamphetamine-induced dysfunction of the BBB, and methamphetamine-induced neuroinflammation.

Of note, the central pathophysiological theory considered in this case is the effect of methamphetamine on the CNS. The CNS is delicate, and normal activity relies on the harmony of electrolytes, excitatory and inhibitory neurotransmitters, and metabolic substrates. One of these crucial neurotransmitters is dopamine, which acts in both the central and peripheral nervous systems to regulate activities related to motor control, cognitive function, reward, and other physiologic functions. Like dopamine, serotonin governs a wide variety of biological processes, including mood, emotion, and hemostasis. Guilarte et al. noted that methamphetamine produces lasting deficits in transporters for dopamine and serotonin in rodents [13]. Additional studies noted that dopamine receptor knockout mice are protected from neurotoxic effects of methamphetamine [14, 15]. These findings could indicate that methamphetamine’s action on dopamine receptors may play a significant role in its direct toxicity on the brain. Seiden and colleagues also suggested that administration of methamphetamine results in the loss of dopamine and norepinephrine in various parts of the brain [16]. Given the importance of dopamine, serotonin, and norepinephrine for normal physiological functions throughout the body, alteration of the balance of these neurotransmitters by methamphetamine could play a role in the encephalopathy seen in this patient.

Another integral part of maintaining CNS homeostasis is the integrity of the BBB structure. Multiple rodent studies suggest that methamphetamine exposure may compromise the function of the BBB [17, 18]. One proposed mechanism for this increased permeability is the alteration of the function of structural proteins. In mice, a single high dose of methamphetamine decreased the expression of tight junction proteins after 24 h post exposure [19]. Tight junctions are one of the primary mechanisms for maintaining the barrier between the systemic vascular system and the CNS. Therefore, compromising the junctional proteins could potentially lead to increased permeability. Bowyer and Ali reported an increase in BBB permeability as evidenced by an increase in IgG immunoreactivity in the medial and ventral amygdala 90 min after administering one dose of methamphetamine to rats [20]. In addition, mice administered methamphetamine had significantly higher levels of fluid and key electrolytes such as sodium, potassium, and chloride in the brain, suggesting development of brain edema. They also demonstrated intense immunostaining for albumin, indicating the breakdown of the BBB [21]. Methamphetamine may also contribute to cerebral edema through damage of the epithelial cells of the choroid plexus, an important structure that helps regulate fluid exchange between the brain and blood vessels [22].

A final pathway proposed in the literature is encephalopathy secondary to inflammatory changes within the brain. It is known that microglia and astrocytes play a key role in brain injury and repair. Microglia monitor neuron firing and synaptic function and thus react rapidly to changes in neuronal activity [23]. However, sustained activation of these cells may lead to damage of brain parenchyma due to excessive production of proinflammatory mediators, including tumor necrosis factor-alpha, interleukin-1B, and interleukin-6 [24]. Studies have revealed that methamphetamine may directly activate microglia in rats and humans [25, 26]. This may lead to brain dysfunction secondary to microgliosis and changes in communication between neurons and glial cells [27–29]. In addition, other models have provided evidence that methamphetamine-induced neurotoxicity can be attenuated through anti-microglial actions [26, 30, 31]. These studies suggest that hyperactivation of microglia and other inflammatory cells within the brain due to acute methamphetamine intoxication can lead to neurodegeneration and adverse behavioral changes like those observed in this case. This mechanism is less likely in this patient since the reversal of patient’s neurological function was faster than expected and no brain edema was noted on the imaging study.

Aside from methamphetamine, other substances such as cocaine and heroin have been reported as causative agents for drug-induced encephalopathy. Cocaine also affects dopamine signaling in the brain through reuptake inhibition. Multiple published reports describe patients developing toxic leukoencephalopathy following cocaine use [32, 33]. In addition, progressive spongiform leukoencephalopathy has also been reported in patients inhaling heated heroin vapor [34].

## Conclusion

There is limited literature available on MIE in the absence of hyperammonemia, making this case unique. Based on a thorough literature review, we propose three possible mechanisms for MIE. The first includes acute disruption of essential neurotransmitters, potentially leading to an acute altered CNS function. Another is methamphetamine-induced disruption of the BBB. Finally, methamphetamine may cause neuroinflammation via activation of microglia and production of pro-inflammatory cytokines leading to brain degeneration. Further research should be conducted to confirm the proposed pathophysiological mechanisms for MIE in the absence of hyperammonemia, which is crucial to optimizing diagnostic and treatment algorithms.

## Data Availability

The datasets generated during and/or analyzed during the current study are available from the corresponding author on reasonable request.
